# Tandem fluorescence and Raman (fluoRaman) characterisation of a novel photosensitiser in colorectal cancer cell line SW480†
†Electronic supplementary information (ESI) available. See DOI: 10.1039/c8an01461b


**DOI:** 10.1039/c8an01461b

**Published:** 2018-11-23

**Authors:** Julia Gala de Pablo, David R. Chisholm, Andreas Steffen, Amanda K. Nelson, Christoph Mahler, Todd B. Marder, Sally A. Peyman, John M. Girkin, Carrie A. Ambler, Andrew Whiting, Stephen D. Evans

**Affiliations:** a Molecular and Nanoscale Physics Group , School of Physics and Astronomy , University of Leeds , Leeds , UK . Email: S.D.Evans@leeds.ac.uk; b LightOx Limited , Wynyard Park House , Wynyard Avenue , Wynyard , Billingham , TS22 5TB , UK; c Department of Chemistry , Durham University , South Road , Durham , DH1 3LE , UK . Email: andy.whiting@durham.ac.uk; d Institut für Anorganische Chemie , Julius-Maximilians-Universität Würzburg , Am Hubland , 97074 Würzburg , Germany; e Welcome Trust Brenner Building , St James's University Hospital , Faculty of Medicine and Health , University of Leeds , Leeds , UK; f Biophysical Sciences Institute , Department of Physics , Durham University , South Road , Durham , DH1 3LE , UK; g Department of Biosciences , Durham University , South Road , Durham , DH1 3LE , UK

## Abstract

The development of new imaging tools, molecules and modalities is crucial to understanding biological processes and the localised cellular impact of bioactive compounds. A small molecule photosensitiser, DC473, has been designed to be both highly fluorescent and to exhibit a strong Raman signal in the cell-silent region of the Raman spectrum due to a diphenylacetylene structure. DC473 has been utilised to perform a range of novel tandem fluorescence and Raman (fluoRaman) imaging experiments, enabling a thorough examination of the compound's cellular localisation, exemplified in colorectal cancer cells (SW480). This multifunctional fluoRaman imaging modality revealed the presence of the compound in lipid droplets and only a weak signal in the cytosol, by both Raman and fluorescence imaging. In addition, Raman microscopy detected the compound in a cell compartment we labelled as the nucleolus, whereas fluorescence microscopy did not detect the fluoRaman probe due to solvatochromatic effects in a local polar environment. This last finding was only possible with the use of tandem confocal Raman and fluorescence methods. By following the approach detailed herein, incorporation of strong Raman functional groups into fluorophores can enable a plethora of fluoRaman experiments, shedding further light on potential drug compound's cellular behaviour and biological activity.

## Abstract 2

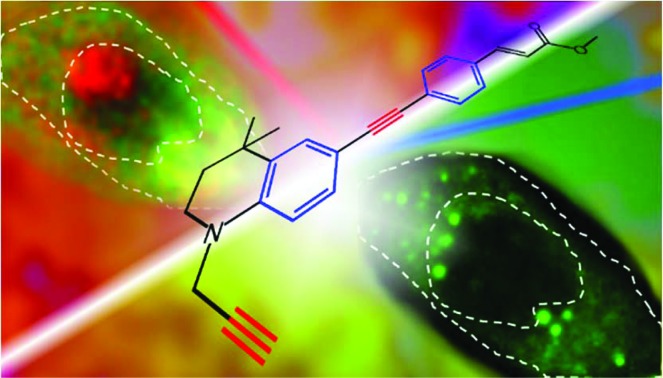
A novel photosensitiser, DC473, designed with solvatochromatic fluorescence and distinct Raman signal, is detected with tandem fluoRaman in SW480 cells.

## Introduction

Imaging and tracing bioactive compounds *in vivo* is essential to understanding their mechanism of action. However, tagging them while retaining their inherent biological activity and function is very difficult, in particular when large fluorescent molecules such as the 238 residue Green Fluorescent Protein (GFP) are employed as labels.^1^ An inherently fluorescent bioactive molecule that does not require labelling is, therefore, highly desirable. However, there are other issues to consider when using fluorescence for quantitative molecular tracking, including photobleaching during the experiment,^1^ solvatochromatic or fluorogenic effects in different locations within the cell and non-linear responses from the fluorophore due to localised concentrations or self-absorption.^2^ For this reason, novel and complementary techniques for the detection and characterisation of a bioactive molecule within a cellular environment are needed.

Raman spectroscopy uses the inelastic scattering of monochromatic light to provide information on the vibrational modes of a sample, allowing the detection of chemical functional groups.^3^ It is a label-free and non-destructive technique, with the ability to diagnose disease states or track the biochemical changes occurring in a cell over time.^4^ Raman spectroscopy can be a valuable tool, not only for imaging a compound within a cell in a semi-quantitative manner, but also for obtaining information on the molecules with which they co-locate.^5^ However, Raman signal intensity is several orders of magnitude weaker than fluorescence or elastic scattering signals making the technique inherently slow and hence, lacking in sufficient sensitivity.^4^

To overcome these drawbacks, and to obtain orthogonal information provided by Raman measurements, multimodal imaging systems can be used.^4^ Fluorescence is usually a problem in Raman spectroscopy, as it can cause significant background signal. Much effort has, therefore, gone into the design of Raman systems to reduce interference from fluorescence.^4^ Since 2003, several groups have adopted the multimodal approach for increasing the tissue-diagnosis sensitivity of Raman probes by combining Raman with tissue auto-fluorescence.^4^,^6^,^7^ Fluorescence tends to be used either as a confirmatory technique for the Raman mapping, or as a quick mapping technique to choose the areas of interest prior to Raman imaging in a large sample.^4^ Some researchers have used both techniques in a complementary manner, such as the work of Carney *et al.* on extracellular vesicles using laser tweezers Raman spectroscopy and fluorescence labelling with CD9+ antibodies.^8^ Raman and fluorescence have also been shown to provide complementary information for studying pH effects of doxorubicin uptake,^9^ for tissue imaging using surface-enhanced Raman scattering (SERS) and fluorescence based endoscopy^10^ and for dual drug release studies, using both surface-enhanced resonance Raman scattering (SERRS) and energy transfer to probe length scales.^11^ Other groups that have combined fluorescence and Raman include Zeng *et al.* who tracked autofluorescence lifetime and stimulated Raman scattering of naturally occurring lignin molecules in poplar cells,^12^ Manen *et al.* tracked both the resonant Raman signal of flavocytochrome b_558_ and the fluorescence of labelled quantum dots in single live neutrophils using the same excitation laser,^13^ and Uzunbajakava *et al.* investigated DNA using spontaneous Raman and two-photon excited fluorescence of the DNA dye Hoechst 33342.^14^ Designing a molecular probe that can be imaged by both tandem fluorescence and Raman scattering could give additional information in the location and environment of the probe. Following this approach, Li *et al.* designed a mitochondrial probe for both stimulated Raman scattering (SRS) and aggregation induced emission (AIE) fluorescence in HeLa cells.^15^ However, to our knowledge, designing a potential chemotherapeutic compound that can be imaged by both fluorescence and specific spontaneous Raman scattering (RS) in tandem (fluoRaman) has not been shown previously.

The vibrational signals associated with chemotherapeutic drugs often occur within the so-called “fingerprint” region of the spectrum, that also contains the vibrational information of both cells and tissue.^16^ Consequently, the reliable and confident analysis of drug distribution in cells can be extremely challenging. Designing bioactive compounds with Raman signatures in the cell-silent region between 1800–2800 cm^–1^ can enable detection of the compound more accurately in cellular samples.^17^ Alkynes are one such example, and typically exhibit a vibrational frequency of *ca.* 2125 cm^–1^ with intrinsically sharp peaks (fwhm ∼14 cm^–1^).^18^ Alkyne labels for Raman spectroscopy were introduced in the last decade by Yamakoshi *et al.*^19^,^20^ and alkyne palettes have been developed by Chen *et al.* using C^13^ and C^12^ isotope combinations^18^ and by Hu *et al.* with their, so called, Carbow.^21^ These probes show significant potential for diagnostic purposes in cancer therapy, using Raman spectroscopy.

Colorectal cancer (CRC) is the third most common cancer worldwide, with 1.4 million new cases diagnosed in 2012, with numbers worldwide predicted to rise to 1.36 million for men and 1.08 million for women by 2035.^22^ Around 50% of the patients develop metastasis to the liver^23^ and resistance to standard chemotherapy.^24^ Novel therapies for the treatment of CRC are, therefore, needed.

**Fig. 1:**
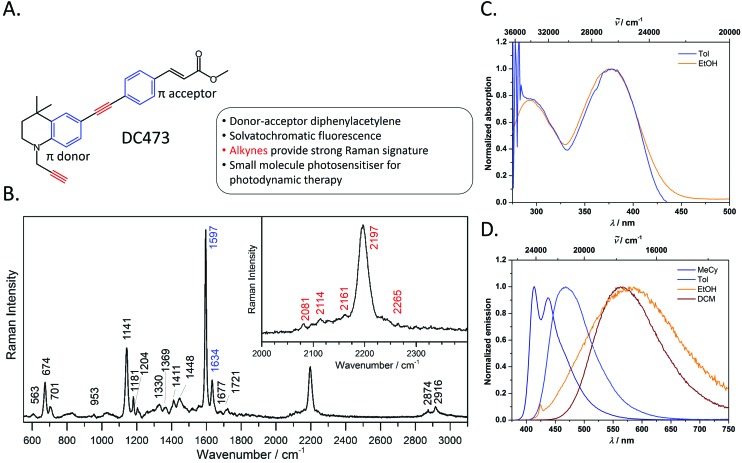
A. Chemical structure and summary of the properties of photosensitiser, DC473. B. Raman spectrum of DC473, in the solid state, taken with the 40× and 785 nm laser excitation; the inset shows the C≡C band. The Raman signals corresponding to the alkyne and phenyl groups are indicated in red and blue on the peak labels, respectively. The main Raman bands tracked in this study are the 1597 cm^–1^ phenyl C=C stretch and the 2197 cm^–1^ C≡C stretch vibrations. C. Absorption and D. emission spectra of DC473 in a variety of solvents MeCy = methylcyclohexane, Tol = toluene and DCM = CH_2_Cl_2_.

Photodynamic therapy utilises photosensitisers to induce the production of large quantities of reactive oxygen species (ROS) for the targeted destruction of diseased tissues, and has been used to treat a variety of cancers.^25^ However, current photosensitisers approved for therapeutic use suffer from a wide variety of issues, including poor pharmacokinetic properties due to their high molecular weight, often porphyrin structures, and ineffective targeting leading to the death of healthy tissue.^25^–^27^ We have developed a new low molecular weight, small photosensitiser, DC473 (Fig. 1A), that elicits cell death through the stimulated production of ROS when activated by UV-A, violet or corresponding near-infrared two-photon wavelengths.^28^ To improve this photosensitising activity, a precise understanding of the compound's cellular localisation is necessary. DC473 exhibits strong solvatochromatic fluorescence and possesses two alkynes suitable for Raman spectroscopy. It was anticipated that this unique combination of structural and photophysical properties would enable a range of novel tandem fluorescence and Raman (fluoRaman) imaging methodologies and experiments. Indeed, herein we report a detailed investigation into the cellular localisation of DC473 in the SW480 CRC cell line using confocal fluorescence and Raman (fluoRaman) microscopy, proving co-location of the compound signal with both techniques, and obtain the Raman signature of the organelles with which it spatially correlates.

## Materials and methods

### General photophysical information

UV-visible absorption spectra were obtained on an Agilent 1100 Series Diode Array spectrophotometer using standard 1 cm path length quartz cells. Excitation and emission spectra were recorded on an Edinburgh Instruments FLSP920 spectrophotometer, equipped with a 450 W xenon arc lamp, double monochromators for the excitation and emission pathways, and a red sensitive photomultiplier (PMT-R928) and a near-IR PMT as detectors, or on a Horiba Jobin–Yvon Fluoromax 3 spectrophotometer with single monochromators for the excitation and emission pathways. The excitation and emission spectra were corrected using the standard corrections supplied by the manufacturers for the spectral power of the excitation source and the sensitivity of the detector. The quantum yields were measured by use of integrating spheres with either the Edinburgh Instruments FLSP920 spectrophotometer or the Horiba Jobin–Yvon Fluoromax 3 spectrophotometer. The luminescence lifetimes were measured using a TCSPC module on an FLSP980 spectrometer equipped with a high-speed photomultiplier tube positioned after a single emission monochromator and operating with pulsed laser diodes (376 or 274 nm, repetition rate 1–5 MHz, pulse width *ca.* 200 ps, instrument response function *ca.* 500 ps). Decays were recorded to 10^4^ counts in the peak channel with a record length of at least 1000 channels. The band-pass of the monochromator was adjusted to give a signal count rate of <20 kHz. Iterative deconvolution of the IRF with one decay function and nonlinear least-squares analysis were used to analyse the data. The quality of all decay fits was judged to be satisfactory, based on the calculated values of the reduced *X*^2^ and Durbin-Watson parameters and visual inspection of the weighted and autocorrelated residuals.

### Cell culture

SW480 cells were provided by Dr N. West from Saint James's University Hospital and were cultured in Dulbecco's modified Eagle medium (DMEM, Gibco) supplemented with 10% fetal bovine serum (Sigma), 2 mM glutamax (Thermo Fisher Scientific) and penicillin 100 units per mL streptomycin 100 μg mL^–1^ (Sigma). All experiments were done with passage numbers below 50. Quartz slides (UQG Optics, 75 × 25 × 1 mm) and coverslips (25.4 × 25.4 × 0.15–0.25 mm Alfa Aesar) were sonicated with acetone (VWR Chemicals), 2–5% decon 90 (VWR Chemicals) and rinsed with milliQ. Hydrogen peroxide 30% (Thermo Fisher) and sulphuric acid >95% (Thermo Fisher) were mixed in a 3 : 7 proportion (Piranha solution) and used to clean the slides for 20 minutes. Slides and coverslips were rinsed and stored in milliQ and dried under a stream of nitrogen immediately before using. Quartz coverslips were coated with poly-l-Lys (Sigma) and basement membrane extract (BME, Cultrex) to increase cell adherence. Treatment with poly-l-lysine was done covering the coverslip with 10 μg mL^–1^ poly-l-lysine solution and incubating for 1 h, storing at 4 °C for 1 week. BME 12–18 mg mL^–1^ was thawed and diluted to ∼150 μg mL^–1^. Coverslips were rinsed with DPBS and BME was added and incubated for 1 h. Cells (viability 96% Trypan blue) were seeded at a concentration of 3.5 × 10^5^ cells per well 2 days in advance. After two days, cells were treated with either DC473 4 μM or the equivalent volume of dimethyl sulfoxide (DMSO, sterile, New England Biolabs) in complete DMEM for 4 hours. After incubation, cells were washed with DPBS and fixed with 4% paraformaldehyde (PFA, Sigma Aldrich) for 10 min. Spacers were prepared using a 50 μm polyethylene terephthalate film (Goodfellow, UK). Cells were measured immediately by bonding the coverslip to a quartz slide, creating a small chamber with the spacers, and sealing the setup with wax to minimize evaporation.

### Confocal Raman spectroscopy and fluorescence

The Raman system used was an inVia Raman confocal inverted microscope (Renishaw) integrated with a Leica DMi8/SP8 laser scanning confocal microscope system. Raman excitation lasers were a 785 nm diode laser (laser power of 45 mW at the sample, intensity of ∼5.7 MW cm^–2^) and a 532 nm laser (laser power of 22 mW on the sample, intensity of ∼2.7 MW cm^–2^) and a 1200 l mm^–1^ and 1800 l mm^–1^ grating for each laser, respectively. Light was collected using a near infrared enhanced CCD array detector (1024 × 256 pixels). Prior to every experiment a spectrum of a silicon sample was collected and the microscope was calibrated to the peak position (520.5 cm^–1^). The cell spectra were obtained using a 100× oil immersion objective (HC PL APO CS2 FWD 0.13 mm NA 1.4) with a slit size of 20 μm. This setting gave an axial resolution (full width half maximum) of 1.7 μm when *Z*-scanning from a silicon sample and tracking the 520.5 cm^–1^ peak. For the individual spectra taken with the 785 nm laser, the exposure time was 1 s with 15 accumulations. For the individual spectra taken with the 532 nm laser exposure time was 1 s with 10 accumulations. For the mapping with the 785 nm laser the step size was 0.7–1 μm and exposure time was 1.8–2 s px^–1^. Confocal fluorescence imaging used excitation at 405 nm and collected emission in the range 432–543 nm with Pinhole size of 1 Airy disc.

### Pre-processing and statistical errors

#### Preprocessing

The spectra obtained were cosmic ray filtered (WiRE® software) and exported as text files for further analysis with in-house built scripts using the Matlab's Statistics and Machine Learning Toolbox (MathWorks). The Matlab functions used are indicated in italics. The silicon peak of a calibration sample was used to calibrate the wavenumber axis of each spectrum. The spectra were gently baseline corrected using the algorithm developed by Koch *et al.* (2016)^29^ with smooth-width of 100, band-width of 600 and 5 iterations, and normalized to the Amide I peak. **Statistical errors**. Unless stated otherwise, all values are expressed ± the standard error calculated as *σ*/√*N*, where *σ* is the standard deviation and N the sample size. **Raman maps**. For all Raman images, contrast was adjusted with the percentile of the data of all pixels from all the maps used using a LUT of 1–99% – where 1% of pixels intensity is saturated at either end of the colour map. A Gaussian filter with radius size of half a pixel was used to smooth the pixel structure of the false colour images. **Intensity images**. Indicated peaks were integrated to the baseline for each of the individual spectra using appropriate peak width, obtaining the integrated intensity. **Principal Component Analysis (PCA)**. The edited data was standardized using Standard Normal Variate and the function pca was used, where the PCs obtained were plotted without any additional normalization. The scores of each pixel were plotted as images.

## Results and discussion

### Synthesis, photophysical properties and Raman characterisation

The photophysical behaviour of diphenylacetylene (tolan) has been widely studied, and the compound's conjugated structure has been extensively modified to tailor its properties towards a variety of applictions.^30^–^39^ By appending strong π-donor (*e.g.* NR_2_) and π-acceptor (*e.g.* CO_2_H, CO_2_R) moieties, the absorption and emission characteristics of these compounds can be significantly bathochromically shifted due to the formation of intramolecular charge transfer (ICT) excited states.^40^,^41^ Diphenylacetylenes are also known to exhibit triple bond stretching vibrations of particularly strong intensity in Raman spectroscopy in comparison to other alkynes.^20^ Thus, diphenylacetylenes potentially represent an ideal structural class for use in fluoRaman imaging.

For this purpose, we designed and synthesised DC473 (Fig. 1A), a substituted diphenylacetylene with a highly lipophilic, electron rich tetrahydroquinoline donor and a methyl cinnamate acceptor (for organic synthetic procedures, see ESI†).^42^ The compound exhibits two strong absorption bands in the UV and violet regions, and a highly solvatochromatic fluorescence emission with quantum yields up to 1.0 in non-polar solvents (Fig. 1C and D and Table 1). In non-polar solvents, DC473 exhibits strong emission in the blue/green region, while in more polar solvents the emission is significantly bathochromically shifted due to stabilisation of the intramolecular charge transfer excited state. The emission is also much weaker in polar solvents such as EtOH. Accordingly, in a cellular context, fluorescence microscopy indicated that DC473 is clearly present in non-polar, membrane-rich environments. However, while the compound was not visible in the cytosol by fluorescence, it would be unwise to infer that the compound was completely absent from this environment.

**Table 1:** Photophysical properties of DC473 in a variety of solvents, where *λ* is the wavelength, *φ* is the quantum yield and *τ* is the fluorescence lifetime

Solvent	*λ*_abs_(max)/nm (*ε*/m^–1^ cm^–1^)	*λ*_em_(max)/nm	*φ*	*τ*/ns
MeCy	—	414, 437	—	—
Toluene	378 (33 200), 292 (24 900)	468	1.00	1.6
EtOH	375, 294	569	0.01	—
CH_2_Cl_2_	—	567	—	—

Hence, we pursued the complementary use of Raman spectroscopy, in tandem with fluorescence, to fully understand and characterise the compound's cellular localisation. Fig. 1B shows the Raman spectrum of DC473 acquired with a 785 nm laser after baseline subtraction, with the characteristic presence of the diphenylacetylene triple bond stretching vibration at 2197 cm^–1^. Alkyne peaks usually appear at *ca.* 2125 cm^–1^;^18^ however, the electron-rich tetrahydroquinoline donor group causes a shift to 2197 cm^–1^.^21^*N*-Propargyl alkynes Raman bands are typically much less intense and of lower wavenumber than their diphenylacetylene counterparts and it is, therefore, likely that this alkyne stretch is present as one of the weaker signals in the 2080–2160 cm^–1^ region. A strong signal assigned as phenyl ring vibrations was also evident at 1597 cm^–1^. Both the phenyl vibrations at 1597 cm^–1^ and the 2197 cm^–1^ diphenylacetylene alkyne peak were used for the detection of the compound *via* Raman spectroscopy in single cells.

### Cellular imaging

**Fig. 2:**
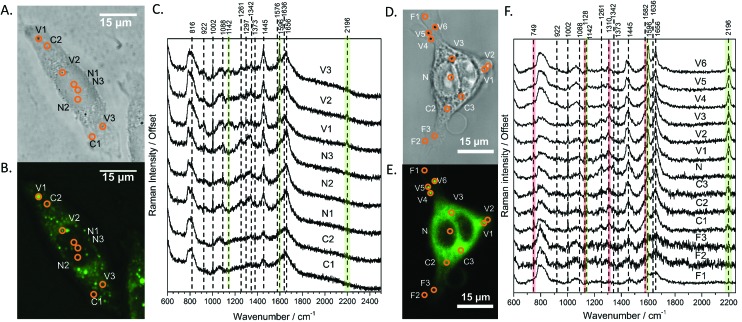
A and D Show the bright field and B and E show the confocal fluorescence images of fixed SW480 cells following incubation with DC473 whose point spectra were measured in C and F, respectively, where V stands for vesicle, N for nucleus, F for filopodia and C for cytosol. C. Raw Raman 785 nm spectra at selected points of the SW480 cell showed in A and B. The green bands show the main peaks associated with the presence of the compound in different cellular locations, with strong presence in vesicles. F. 532 nm Raman baseline subtracted spectrum of selected points of the SW480 cell showed in D and E. The green bands show the main DC473 peaks, showing a similar trend to Fig. 1B. Measurements in the cytosol show weak signal of DC473 and resonant Raman bands of cytochrome c, indicated with red bands.

With the fluorescence and Raman signatures of DC473 understood, we next sought to characterise the compound's cellular localisation and behaviour using the primary adenocarcinoma colorectal cancer cell line, SW480. First, the Raman spectra were acquired with a 785 nm laser at individual points in fixed SW480 cells that had been incubated with DC473, and compared with the confocal fluorescence images of the same cells. Fig. 2C shows high-quality raw Raman spectra taken from different parts of a fixed cell. DC473 accumulation was detected when measuring in vesicles and in membranes, but not in the nucleus. The fluorescence image in Fig. 2B shows accumulation of the compound mainly in vesicles and weakly in the cytosol, but not in the nucleus. Raman measurements in the indicated points (V1–3) confirmed the presence of DC473 in particles which correlate with the strong 1455 cm^–1^ band associated with lipids, indicating that they were likely lipid droplets, previously described in this cell line.^43^,^44^ Cytosol measurements showed weak signals of DC473, in agreement with the fluorescence characterisation. Single point measurements in the nucleus showed only a very weak signal from the compound.

When exciting with the 532 nm laser, cytochrome c, a small, soluble heme-protein localised in mitochondria, shows a resonant Raman signal.^45^,^46^ Fig. 2D and E show the bright field and confocal fluorescence images of an SW480 cell following incubation with DC473. Fig. 2F shows individual points probed with RS with a 532 nm laser in the same cell in vesicles, the nucleus, cytosol and filopodia. Similar to the results obtained with the near-infrared excitation, DC473 was detected strongly in vesicles and weakly in the cytosol. The cytosol regions examined exhibited the characteristic 749, 1128, 1310 and 1582 cm^–1^ cytochrome peaks, indicating that the DC473 in this compartment could be localized near, or inside, the mitochondria. Mitochondria are an important target for photodynamic therapy agents and it is, therefore, important to understand the behaviour of DC473 with respect to co-localisation with the mitochondria signal. Ideally, higher resolution studies should be undertaken to confirm this co-localisation. However, given the large membranous area associated with the mitochondria and the ability of DC473 to accumulate in lipid rich environments, this seems reasonable. Indeed, given that the DC473 signal is not resonantly enhanced whilst that of the cytochrome c is, would imply significant accumulations of DC473 in the mitochondria, perhaps as high as 3–4 orders of magnitude greater than that of cytochrome c.

### Mapping of DC473 accumulation using confocal fluorescence and Raman spectroscopy

**Fig. 3:**
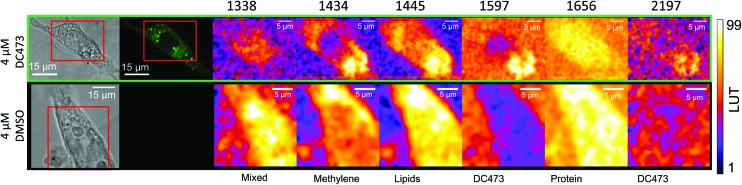
Peak integrated intensity maps for SW480 fixed cells either incubated with DC473 or with an equivalent concentration of DMSO as a control. The 1597 and 2197 cm^–1^ peaks show DC473 accumulation mainly in lipid droplets, as indicated by the correlation with the 1445 cm^–1^ intensity map mainly due to lipid vibrations. The 1338 cm^–1^ intensity map shows mixed contributions of proteins and nucleic acids, and the 1656 cm^–1^ map shows Amide I intensity as a map of proteins. The 1455 and 1434 cm^–1^ maps mainly show contributions of lipids.

Raman mapping was undertaken on fixed SW480 cells incubated with the compound, or with an equivalent concentration of DMSO, as a control (Fig. 3) using 785 nm excitation. This excitation was chosen due to a small but non-zero overlap of the fluorescence excitation spectrum of the molecule with the 532 nm excitation line. Bright field and fluorescence images for the DC473-treated cell are shown together with the Raman maps associated with the main spectral peaks. The 1338 cm^–1^ band, usually assigned as a mixed contribution of CH_2_ wagging, C–O stretching, phenyl stretching and N–H deformation,^47^–^49^ appears most strongly here in the nucleus. The 1434 cm^–1^ band associated with methylene stretching^50^,^51^ shows a strong contribution in lipid droplets rich regions and exhibits a strong correlation with the 1455 cm^–1^ band associated with lipids and fatty acids^50^ as well as the 1597 cm^–1^ phenyl peak associated with DC473. The 1656 cm^–1^ Amide I band^50^ due to proteins is evident over the whole cell area. The two main DC473 peaks, 1597 cm^–1^ (phenyl) and 2197 cm^–1^ (alkyne), show a strong correlation with the lipid peaks and a weaker contribution in the cytosol, indicating localisation of the compound in lipid-rich environments. Whilst the 1597 cm^–1^ DC473 peak is very strong and provides good contrast in the Raman images, its presence on the side of the Amide I peak makes it difficult to separate from that of protein. In contrast, the 2197 cm^–1^ band, whilst weaker, occurs in the ‘silent’ region of the spectrum giving unequivocal detection of the compound.

**Fig. 4:**
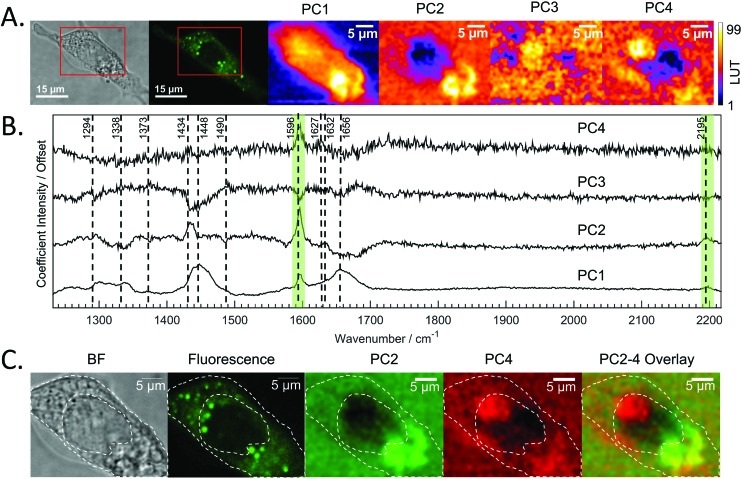
A. Bright field and confocal fluorescence images of the fixed SW480 cell incubated with DC473. The maps of each of the scores of the 4 main PCs are shown. B. Coefficients for the first 4 PCs whose scores are mapped in A. C. Bright field, fluorescence and score maps of PCA coefficients with DC473 associated Raman bands. The cell main features have been overlaid with the images as a visual aid. The presence of PC4 suggests that Raman could be detecting presence of DC473 in a region in the nucleus that does not show in the fluorescence image.

PCA analysis of one of the maps of a cell incubated with DC473 is shown in Fig. 4A, along with the coefficients of these components in Fig. 4B. PC1 shows mainly average cell signal with DC473 contribution, where the whole cell shows higher scores than the background and thus gives similar information to the fluorescence image. PC3 peaks in the nucleus, with negative contributions for both DC473 and the broad lipid band around 1448 cm^–1^. Similarly to the intensity maps, PC2 shows positive contributions for the region around 1338 cm^–1^. PC2 and PC4 show the main DC473-specific components. PC2 shows very strong DC473 presence and a positive 1434 cm^–1^ methylene band indicating accumulation in the elongated structures in the region below the nucleus in Fig. 4A/C.

PC4 shares some similarities with PC2, but with lower DC473 content and with stronger contribution of a band at 1627 cm^–1^. This component shows the presence of the compound in different regions of the cell that do not correlate with the fluorescence signal, as shown in Fig. 4. The PC4 component indicates the presence of DC473 in a compact area inside the nucleus, suggesting that a low concentration of the compound could be present inside the nucleolus. The absence of fluorescence in this region might be explained by a solvatochromatic reduction in quantum yield when the compound is localised in a more polar environment. Hence, the use of fluoRaman microscopy can not only identify the presence of the compound in different cell compartments but also provide information about the polarity of the environment of the compound.

## Conclusions

A continuing and major requirement in the life sciences is for small molecules that can be specifically targeted to sites within cells, which report back on local conditions within the cell, and can then be externally activated to alter cell activity using a minimally invasive method, such as light. We have designed and synthesised a novel photosensitiser, DC473, which is both fluorescent and Raman active in the silent region of the spectra and can, therefore, be tracked within the cell, with the additional advantage of its solvatochromatic fluorescence properties changing due to local conditions. By exploiting the unique structural and spectroscopic properties of DC473, we have utilised fluoRaman microscopy to characterise and understand the cellular localisation of the compound in CRC SW480 cells. These novel imaging experiments have allowed us to detect the compound mainly in lipid droplets and in the cytosol, where it correlates with the resonant Raman signal of cytochrome C from mitochondria, suggesting that it could be located in or near this cellular compartment. Furthermore, we have acquired maps from single SW480 CRC fixed cells where Raman imaging strongly detects the compound in lipid droplet rich areas and weakly in the cytosol. However, further analysis using PCA also shows weak signal for DC473 within the nucleus, suggesting it could also be present in the nucleolus, where it does not show fluorescence due to solvatochromatic effects in a more polar environment. The detection of the compound in the latter, and its polar characteristic, would not have been possible using either of the two techniques used here on their own, *i.e.*, a fluoRaman confocal imaging approach offers a clear advantage for studying this kind of reporter molecules. We anticipate that, by application of the types of chemical design principles we have employed herein, combined with the novel fluoRaman methods, this type of imaging has significant potential in a host of cellular and tissue imaging contexts. This approach can likely be readily applied to a range of other imaging probes and drugs, potentially providing new insights into the cellular behaviour of not only these compounds, but enabling new and unexplored molecular probing methods to be developed.

## Conflicts of interest

AW and CAA are shareholders and directors of LightOx Ltd, a company licensed to pursue commercial applications for the novel compound described in this manuscript.

## Supplementary Material

Supplementary information
