# Supplementary material for: Tandem fluorescence and Raman (fluoRaman) characterisation of a novel photosensitiser in colorectal cancer cell line SW480
Source: Analyst. 2018 Nov 23;143(24):6113–20. doi: 10.1039/c8an01461b (PMC6336151; doi:10.1039/c8an01461b)
Supplement: Supplementary file 1 [file AN-143-C8AN01461B-s001.pdf]

Electronic Supplementary Material (ESI) for Analyst.
This journal is © The Royal Society of Chemistry 2018

SUPPLEMENTARY INFORMATION

Tandem fluorescence and Raman (fluoRaman) characterisation of a novel photosensitiser in colorectal cancer cell line SW480

Julia Gala de Pablo,[a] David R. Chisholm,[bc] Andreas Steffen,[e] Amanda K. Nelson,[e] Christoph Mahler,[e] Todd B. Marder,[e] Sally A. Peyman,[ad] John M. Girkin,[f] Carrie A. Ambler,[bg] Andrew Whiting,[bc] Stephen D. Evans[ad]

a. *Molecular and Nanoscale Physics Group, School of Physics and Astronomy, University of Leeds, Leeds, UK.*
b. *LightOx Limited, Wynyard Park House, Wynyard Avenue, Wynyard, Billingham, TS22 5TB, UK.*
c. *Department of Chemistry, Durham University, South Road, Durham, DH1 3LE, UK.*
d. *Welcome Trust Brenner Building, St James's University Hospital, Faculty of Medicine and Health, University of Leeds, Leeds, UK.*
e. *Institut für Anorganische Chemie, Julius-Maximilians-Universität Würzburg, Am Hubland, 97074 Würzburg, Germany.*
f. *Biophysical Sciences Institute, Department of Physics, Durham University, South Road, Durham, DH1 3LE, UK.*
g. *Department of Biosciences, Durham University, South Road, Durham, DH1 3LE, UK.*

Table of contents

1. General synthetic information **P2**

2. Synthetic procedures and characterisation **P3-5**

2. ^{1}H and ^{13}C NMR spectra **P6-10**

3. References **P11**

2. General synthetic information

Reagents were purchased from Sigma-Aldrich, Acros Organics, Alfa-Aesar and Fluorochem. Reagents were purified, if required, by recrystallisation or distillation/sublimation under vacuum. Solvents were used as supplied from Fisher Scientific or Sigma Aldrich, and dried before use, if required, with appropriate drying agents or using an Innovative Technologies Inc. Solvent Purification System. Thin layer chromatography (TLC) was conducted using Merck Millipore silica gel 60G F254 25 glass plates and/or TLC-PET foils of aluminium oxide with fluorescent indicator 254 nm (40 × 80 mm) with visualisation by UV lamp or appropriate staining agents. Flash column chromatography was performed using SiO2 from Sigma-Aldrich (230-400 mesh, 40-63 µm, 60 Å), or activated neutral aluminium oxide (Alumina) from Sigma-Aldrich, and monitored using TLC. Sublimation/distillation was performed using a Buchi Glass Oven B-585 Kugelrohr operating at a pressure between 0.2-2.0 Torr. NMR spectra were recorded using Varian VNMRS-700, Varian VNMRS-600, Bruker Avance-400 or Varian Mercury-400 spectrometers operating at ambient probe temperature. NMR peaks are reported as singlet (s), doublet (d), triplet (t), quartet (q), broad (br), septet (sept), combinations thereof, or as a multiplet (m), with reference to the following deuterated solvent signals: CDCl3 (^{1}H = 7.26 ppm, ^{13}C = 77.23 ppm), $(CD_3)_2SO$ (^{1}H = 2.50 ppm, ^{13}C = 39.50 ppm). ESMS was performed by the Durham University departmental service using a TQD (Waters Ltd., UK) mass spectrometer with an Acquity UPLC (Waters Ltd., UK), and accurate mass measurements were obtained using a QtoF Premier mass spectrometer with an Acquity UPLC (Waters Ltd., UK). ASAP measurements were performed using an LCT Premier XE mass spectrometer and an Acquity UPLC (Waters Ltd., UK). GCMS was performed using a QP2010-Ultra (Shimadzu) GCMS. IR spectra were recorded using a Perkin Elmer FTIR spectrometer. Melting points were obtained using a Gallenkamp melting point apparatus and are uncorrected. Elemental analysis was conducted by the Durham University departmental service using an Exeter Analytical CE-440 analyser.

2. Synthesis

2.1 Synthesis description

DC473 was synthesised via a convergent strategy involving the coupling of donor 6-iodo-tetrahydroquinoline **4** and acceptor alkyne **5**. Tetrahydroquinoline **4** (Figure 1) was synthesised by an initial amide reduction of the previously reported tetrahydroquinolin-2-one **2**[1] using borane dimethylsulphide, followed by alkylation with TMS-protected propargyl bromide **1** to give protected 6-iodo-tetrahydroquinoline **4**. Sonogashira coupling (Figure 2) with the previously reported alkyne **5**[2] provided protected diphenylacetylene **6**, and removal of the TMS protecting group using TBAF afforded the desired photosensitiser, **DC473**.

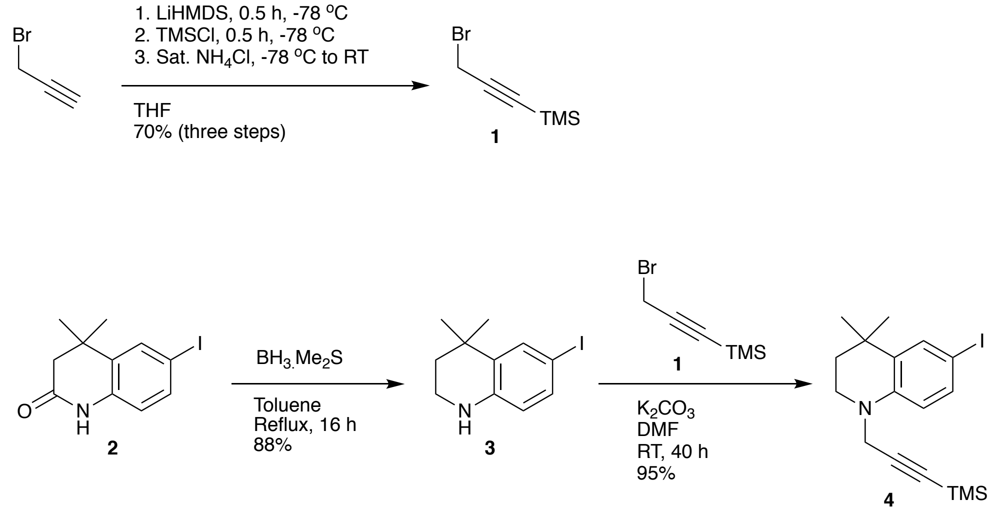

Figure 1: Synthesis of TMS-protected bromide **1,** and protected *N*-propargyl 6-iodo-tetrahydroquinoline **4**.

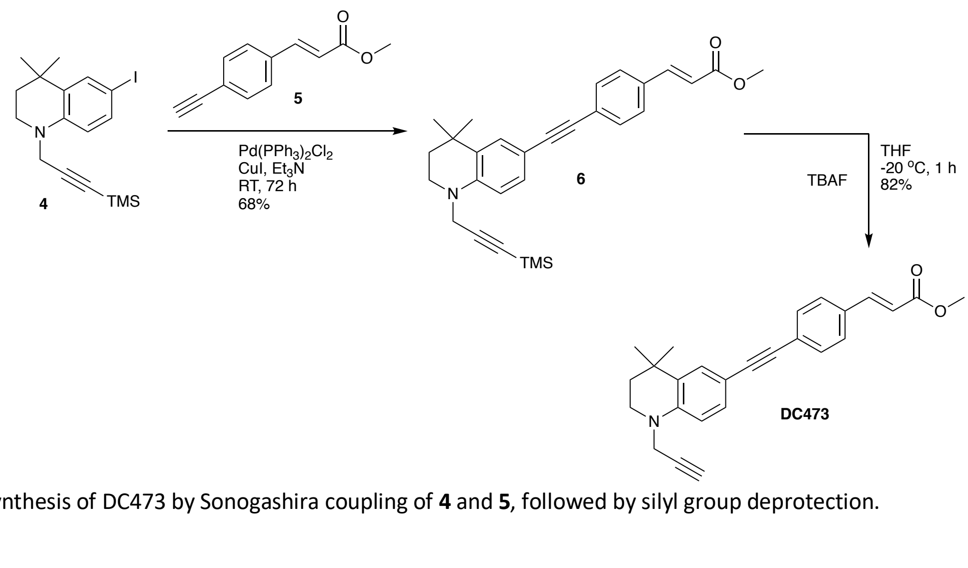

Figure 2: Synthesis of DC473 by Sonogashira coupling of **4** and **5**, followed by silyl group deprotection.

2.2 Experimental procedures and characterisation

(3-Bromoprop-1-yn-1-yl)trimethylsilane, 1

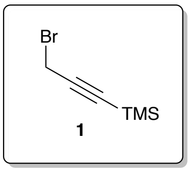

A solution of propargyl bromide (80% in toluene, 6.69 mL, 60.0 mmol) in THF (75 mL) was cooled to -78 °C under Ar. Lithium bis(trimethylsilyl)amide (10.37 g, 62.0 mmol) was added under Ar, and the solution then stirred for 0.5 h at -78 °C. Chlorotrimethylsilane (10.15 mL, 80.0 mmol) was then added dropwise, and the solution stirred for 0.5 h, whereupon sat. NH_4Cl (30 mL) was added, and the solution then warmed to RT. The solution was diluted with EtOAc, washed with brine, dried ($MgSO_4$) and evaporated to give a crude oil. This was purified by SiO_2 chromatography (hexane as eluent), and then further purified by Kugelrohr distillation (70-90 °C, ambient pressure) to give compound **1** as a clear oil (8.09 g, 70%): ^{1}H NMR (400 MHz; $CDCl_3$) δ 0.18 (s, 9H), 3.91 (s, 2H); ^{13}C{^{1}H} NMR (101 MHz; $CDCl_3$) δ -0.1, 14.9, 92.5, 100.2; IR (ATR) ν_{max}/cm^{-1} 2960w, 2906w, 2180w, 1251m, 837s; MS (EI): *m/z* = 174.9 [M]$^{+}$.[3]

Synthesis of tetrahydroquinolin-2-one 2

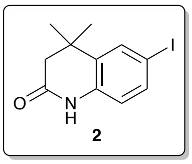

Full details for the synthesis of tetrahydroquinolin-2-one **2** are available in a previous report.[1]

6-Iodo-4,4-dimethyl-1,2,3,4-tetrahydroquinoline, 3

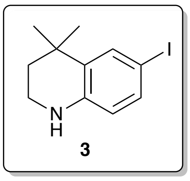

To a solution of compound **2** (4.00 g, 13.28 mmol) in anhydrous toluene (30 mL) under Ar was added borane dimethyl sulphide complex (2.0 M in THF, 8.4 mL, 16.8 mmol) dropwise and the resultant solution stirred at reflux for 16 h. The solution was cooled to RT, 10% Na_2CO_3 (25 mL) added and the solution stirred for 0.5 h. The solution was then diluted with EtOAc, washed with brine, dried ($MgSO_4$) and evaporated to give a crude colourless oil. This was purified by SiO_2 chromatography (hexane:EtOAc, 9:1, with 1% Et_3N, as eluent) to give compound **3** as a colourless oil (3.36 g, 88%): ^{1}H NMR (700 MHz; $CDCl_3$) δ 1.27 (s, 6H), 1.70 (t, *J* = 5.9 Hz, 2H), 3.30 (t, *J* = 5.9 Hz, 2H), 3.93 (br, 1H), 6.24 (d, *J* = 8.4 Hz, 1H), 7.19 (dd, *J* = 8.4, 2.1 Hz, 1H), 7.41 (d, *J* = 2.1 Hz, 1H); ^{13}C{^{1}H} NMR (176 MHz; $CDCl_3$) δ 30.9, 32.0, 36.8, 38.4, 77.7, 116.5, 133.1, 135.1, 135.3, 143.4; IR (ATR) ν_{max}/cm^{-1} 3400br, 2956w, 2927w, 2862w, 1589m, 1524m, 1492s, 1352m, 1282s, 804s; MS(ES): *m/z* = 288.0 [M+H]$^{+}$; HRMS (ES) calcd. for $C_{11}H_{15}NI$ [M+H]$^{+}$: 288.0246, found 288.0242.

6-Iodo-4,4-dimethyl-1-[3-(trimethylsilyl)prop-2-yn-1-yl]-1,2,3,4-tetrahydroquinoline, 4

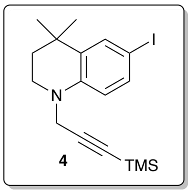

K_2CO_3 (1.39 g, 10.08 mmol) was added to a solution of compound **3** (2.07 g, 7.20 mmol) in anhydrous DMF (25 mL) under Ar and the resultant slurry was stirred for 1 h. Compound **1** (1.65 mL, 10.08 mmol) was added, and the solution was stirred at RT for 72 h. The solution was diluted with H_2O, and extracted with EtOAc (3×). The organics were washed with sat. NH_4Cl, H_2O and brine, dried ($MgSO_4$) and evaporated to give a crude yellow oil. This was purified by SiO_2 chromatography (hexane:EtOAc, 96:4, with 1% Et_3N as eluent) to give compound **4** as a light yellow oil (2.71 g, 95%): ^{1}H NMR (700 MHz; $CDCl_3$) δ 0.13 (s, 9H), 1.26 (s, 6H), 1.74-1.78 (m, 2H), 3.27-3.31 (m, 2H), 3.99 (s, 2H), 6.49 (d, *J* = 8.7 Hz, 1H), 7.33 (dd, *J* = 8.7, 2.2 Hz, 1H), 7.42 (d, *J* = 2.2 Hz, 1H); ^{13}C{^{1}H} NMR (176 MHz; $CDCl_3$) δ 0.2, 30.7, 32.4, 36.9, 42.0, 45.6, 78.8, 89.0, 101.4, 114.7, 134.6, 135.5, 135.5, 143.3; IR (ATR) ν_{max}/cm^{-1} 2958w, 2925w, 2856w, 2169w, 1584m, 1491m, 1332m, 1248m, 838s; MS(ES): *m/z* = 398.5 [M+H]$^{+}$; HRMS (ES) calcd. for $C_{17}H_{25}SiNI$ [M+H]$^{+}$: 398.0801, found 398.0797.

Synthesis of alkyne 5

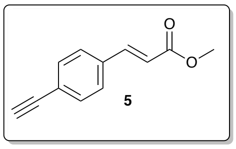

Full details for the synthesis of acceptor alkyne **5** are available in a previous report.[2]

Methyl (2*E*)-3-[4-(2-{4,4-dimethyl-1-[3-(trimethylsilyl)prop-2-yn-1-yl]-1,2,3,4-tetrahydroquinolin-6-yl}ethynyl)phenyl]prop-2-enoate, 6

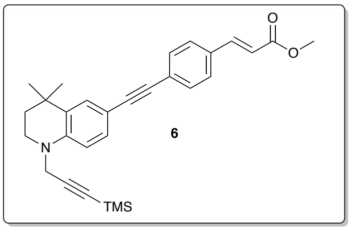

Compound **4** (1.61 g, 4.05 mmol) was dissolved in Et_3N (35 mL), and the resultant solution was degassed by sonication under vacuum, before the atmosphere was replaced with Ar (5×). $Pd(PPh_3)_2Cl_2$ (0.28 g, 0.405 mmol), CuI (0.077 g, 0.405 mmol) and compound **5** (0.79 g, 4.25 mmol) were then added under Ar. The resultant suspension was stirred at RT for 72 h. The suspension was diluted with hexane and passed through a thin Celite®/SiO_2 plug (eluting with hexane, then Et_2O). The extracts were washed with sat. NH_4Cl (3×) and brine, dried ($MgSO_4$) and evaporated to give a crude yellow oil. This was purified by SiO_2 chromatography (hexane:EtOAc, 9:1, with 1% Et_3N as eluent) to give compound **6** as a thick yellow oil (1.25 g, 68%): ^{1}H NMR (700 MHz; $CDCl_3$) δ 0.12 (s, 9H), 1.30 (s, 6H), 1.77-1.81 (m, 2H), 3.33-3.37 (m, 2H), 3.81 (s, 3H), 4.05 (s, 2H), 6.43 (d, *J* = 16.0 Hz, 1H), 6.68 (d, *J* = 8.6 Hz, 1H), 7.27 (dd, *J* = 8.6, 2.0 Hz, 1H), 7.38 (d, *J* = 2.0 Hz, 1H), 7.46-7.52 (m, 4H), 7.67 (d, *J* = 16.0 Hz, 1H); ^{13}C{^{1}H} NMR (176 MHz; $CDCl_3$) δ 0.2, 30.5, 32.3, 36.9, 42.0, 45.7, 51.9, 87.3, 89.0, 93.8, 101.3, 110.5, 112.2, 118.0, 126.5, 128.2, 129.6, 130.8, 131.8, 132.5, 133.4, 144.1, 144.4, 167.6; IR (ATR) ν_{max}/cm^{-1} 3042w, 2957w, 2927w, 2858w, 2195w, 1718s, 1595s, 1515s, 1324s, 1170s, 842s; MS(ES): *m/z* = 456.2 [M+H]$^+$; HRMS (ES) calcd. for $C_{29}H_{34}NO_2Si$ [M+H]$^+$: 456.2359, found 456.2345.

Methyl (2*E*)-3-(4-{2-[4,4-dimethyl-1-(prop-2-yn-1-yl)-1,2,3,4-tetrahydroquinolin-6-yl]ethynyl}phenyl)prop-2-enoate, DC473

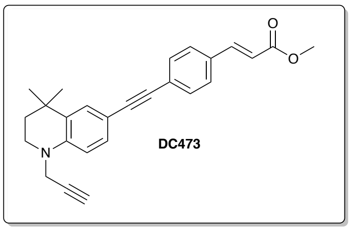

Compound **6** (1.20 g, 2.64 mmol) was dissolved in anhydrous THF (30 mL), and cooled to -20 °C. Tetrabutylammonium fluoride (1.0 M in THF, 2.90 mL, 2.90 mmol) was then added dropwise and the resultant solution stirred at -20 °C for 1 h, after which H_2O was added, and the solution extracted with EtOAc (3×). The organics were washed with brine, dried ($MgSO_4$) and evaporated to give a crude solid. This was purified by SiO_2 chromatography (hexane:EtOAc, 8:2, with 1% Et_3N as eluent) to give DC473 as a yellow oil that slowly crystallised to give a yellow solid (0.83 g, 82%): m.p. = 101-102 °C; ^{1}H NMR (400 MHz; $CDCl_3$) δ 1.30 (s, 6H), 1.76-1.83 (m, 2H), 2.17-2.19 (m, 1H), 3.34-3.38 (m, 2H), 3.81 (s, 3H), 4.05 (d, *J* = 2.4 Hz, 2H), 6.43 (d, *J* = 16.0 Hz, 1H), 6.68 (d, *J* = 8.6 Hz, 1H), 7.28 (dd, *J* = 8.6, 1.9 Hz, 1H), 7.39 (d, *J* = 1.9 Hz, 1H), 7.43-7.54 (m, 4H), 7.67 (d, *J* = 16.0 Hz, 1H); ^{13}C{^{1}H} NMR (101 MHz; $CDCl_3$) δ 30.7, 32.3, 36.9, 41.0, 45.9, 51.9, 72.0, 79.3, 87.3, 93.7, 110.9, 111.9, 118.0, 126.5, 128.2, 129.8, 130.8, 131.9, 132.6, 133.5, 143.9, 144.4, 167.6; IR (ATR) ν_{max}/cm^{-1} 3288w, 2954w, 2927w, 2861w, 2194m, 1716s, 1595s, 1515s, 1496m, 1324s, 1170s, 830s; MS(ES): *m/z* = 384.4 [M+H]$^+$; HRMS (ES) calcd. for $C_{26}H_{26}NO_2$ [M+H]$^+$: 384.1964, found 384.1963; Anal. Calc. for $C_{26}H_{25}NO_2$: C, 81.43; H, 6.57; N, 3.65%. Found: C, 81.31; H, 6.54; N, 3.69%.

3. ^{1}H and ^{13}C NMR spectra

(3-Bromoprop-1-yn-1-yl)trimethylsilane, 1

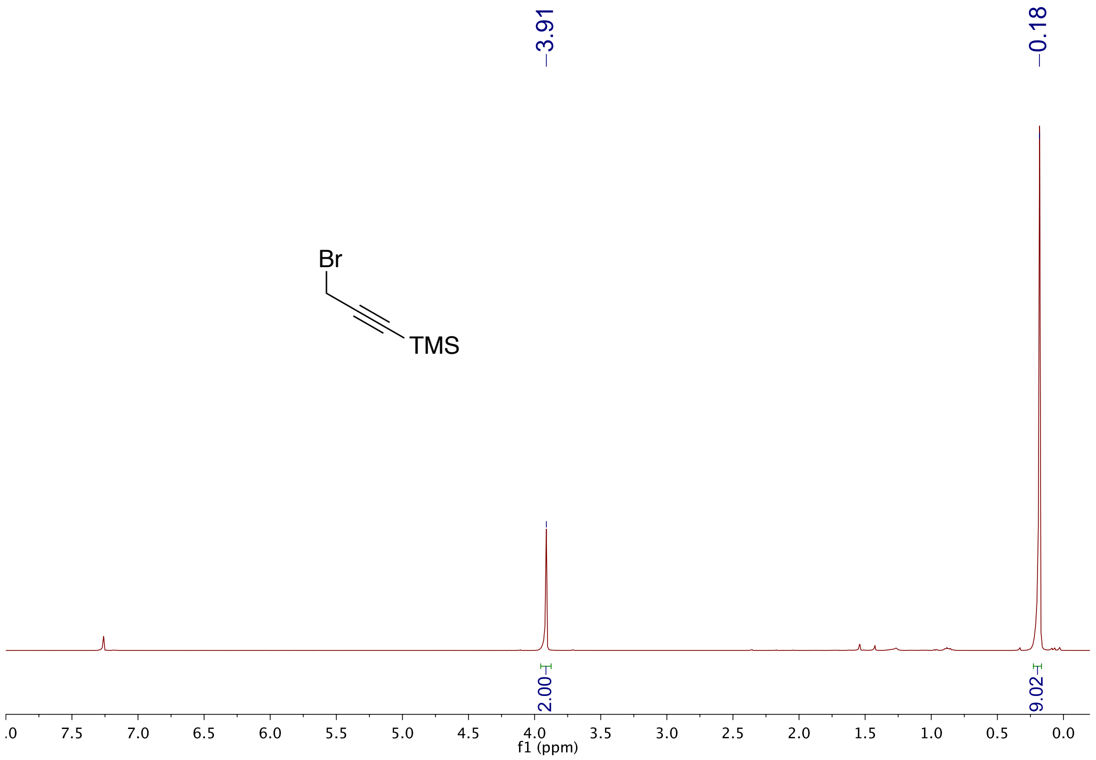

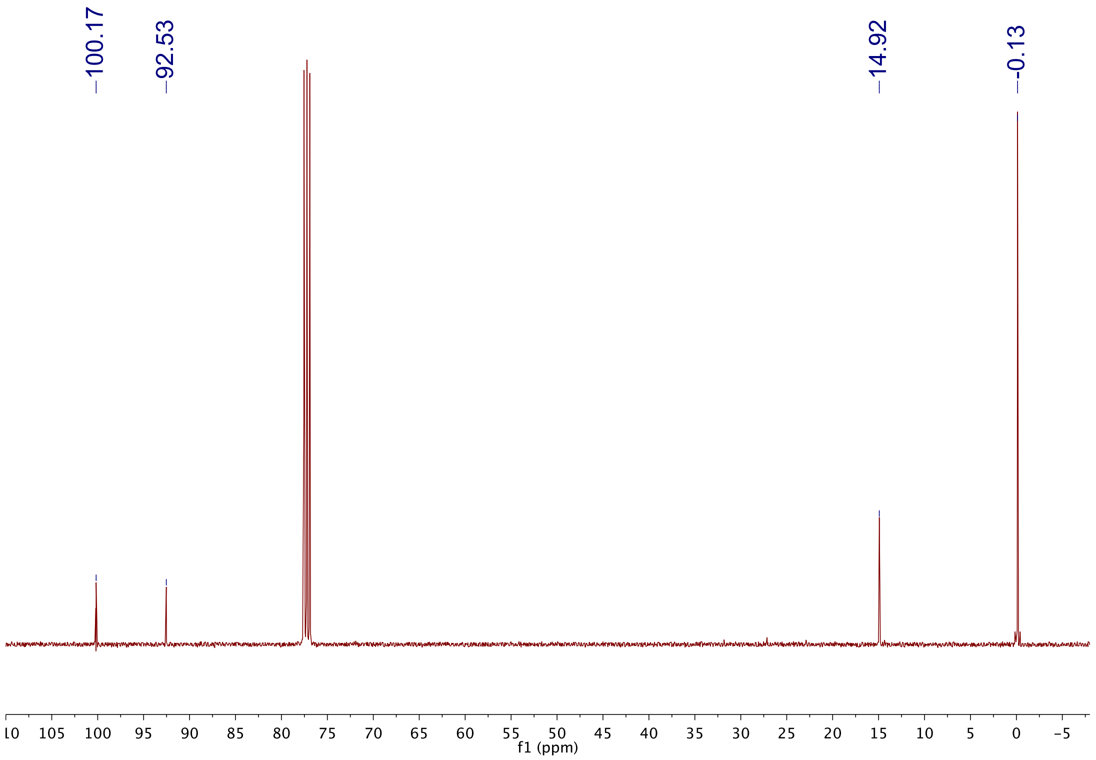

6-Iodo-4,4-dimethyl-1,2,3,4-tetrahydroquinoline, 3

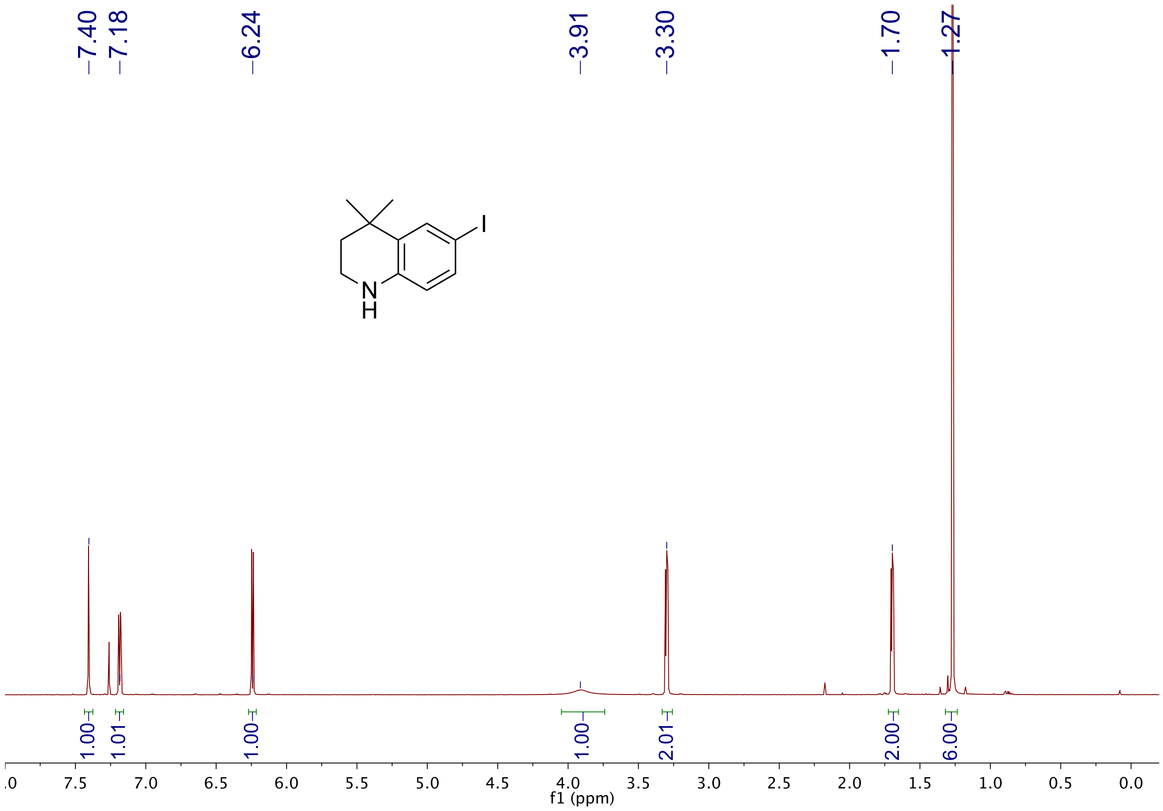

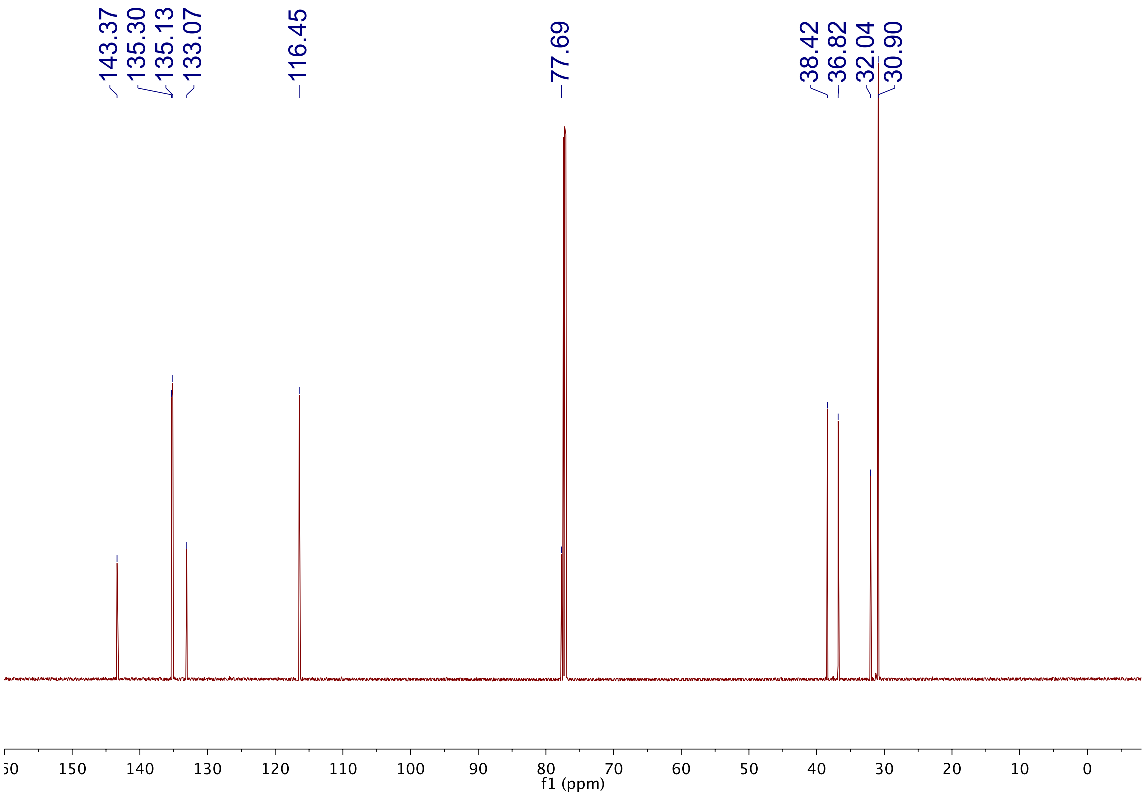

6-Iodo-4,4-dimethyl-1-[3-(trimethylsilyl)prop-2-yn-1-yl]-1,2,3,4-tetrahydroquinoline, 4

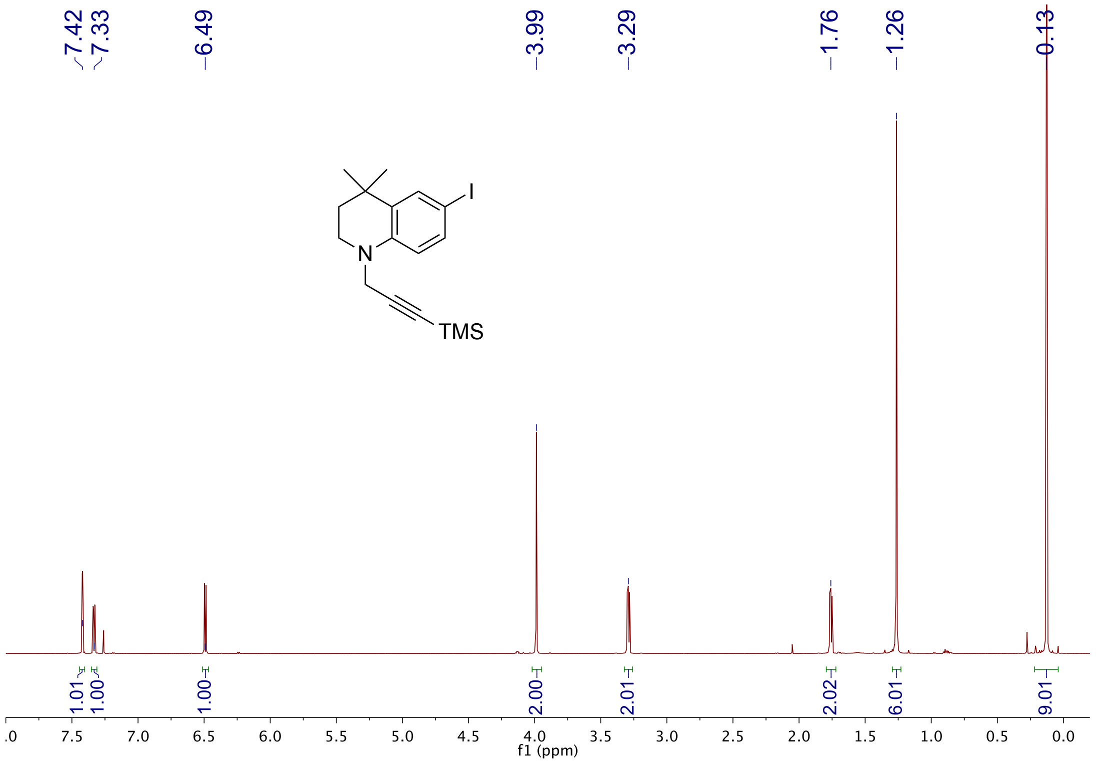

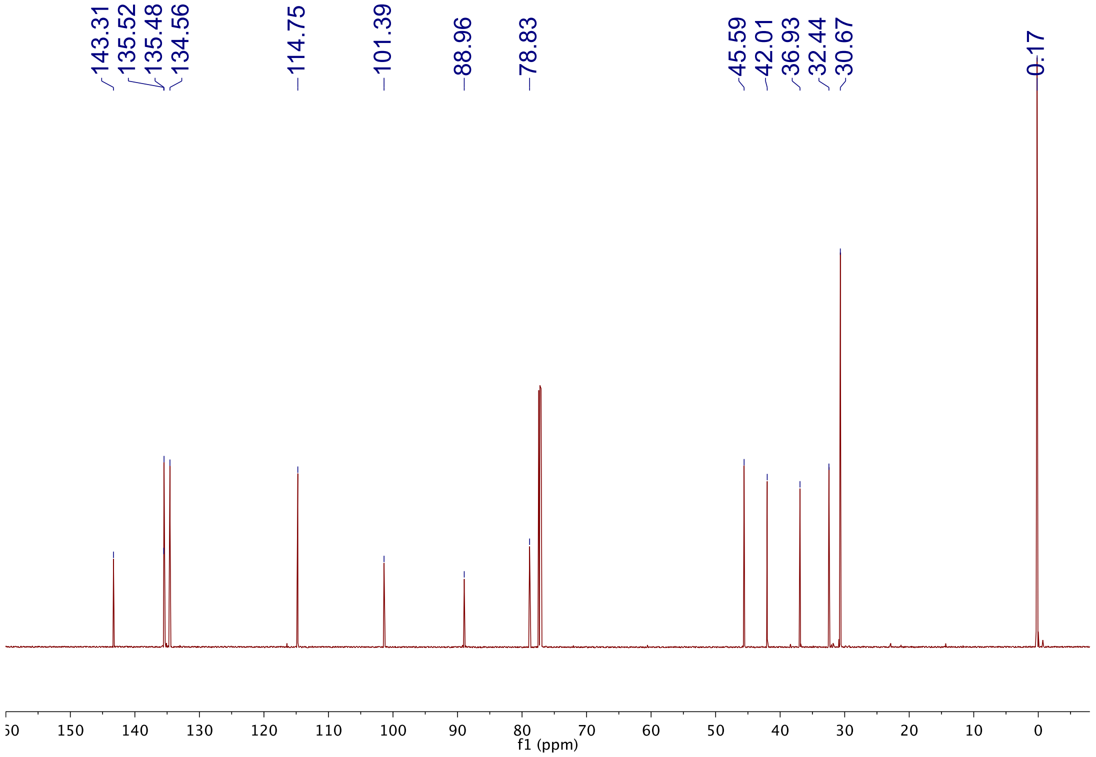

Methyl (2*E*)-3-[4-(2-{4,4-dimethyl-1-[3-(trimethylsilyl)prop-2-yn-1-yl]-1,2,3,4-tetrahydroquinolin-6-yl}ethynyl)phenyl]prop-2-enoate, 6

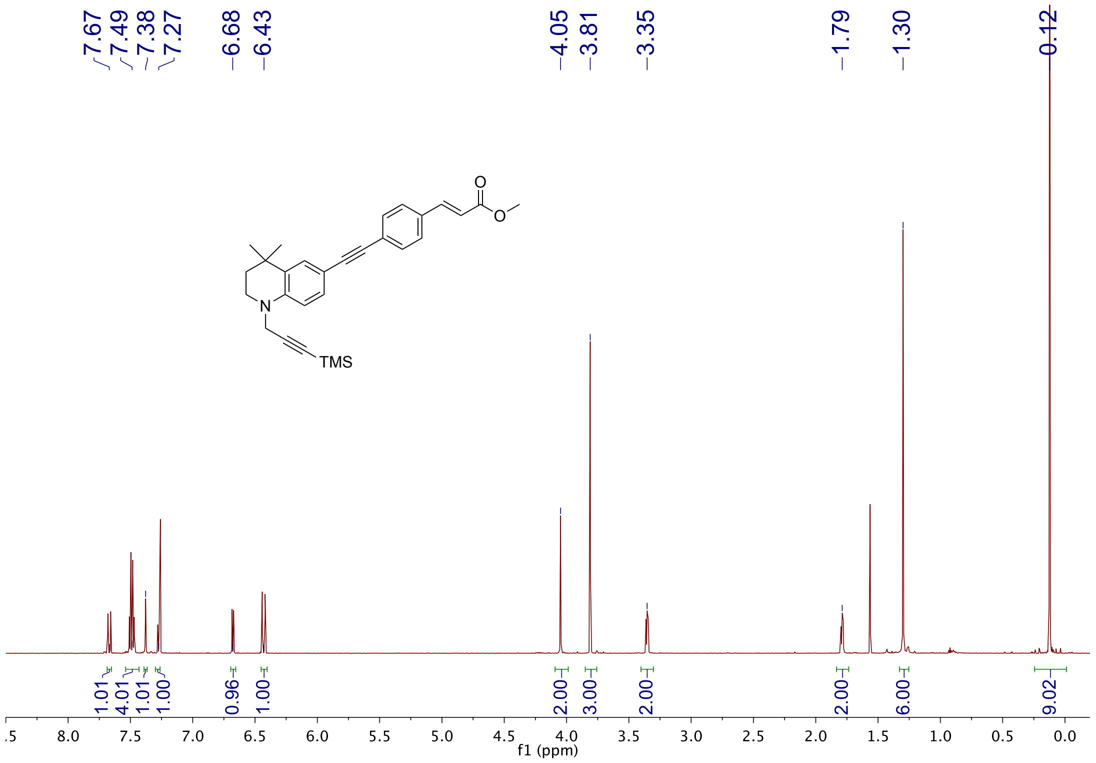

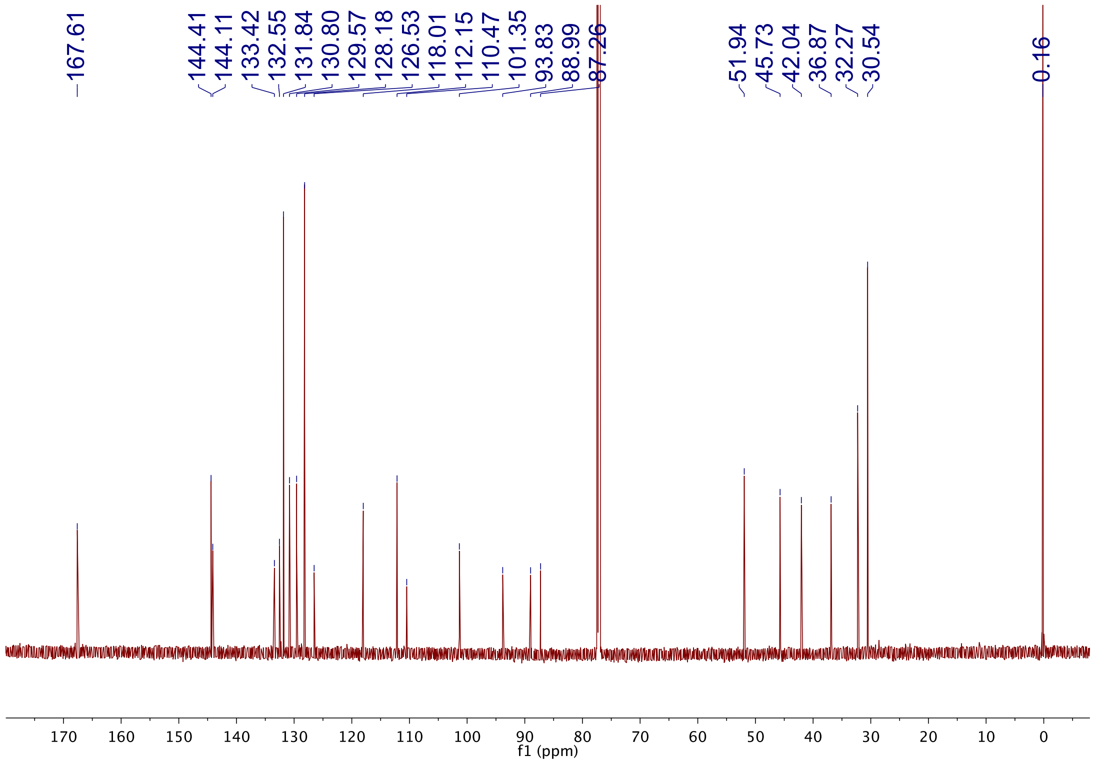

Methyl (2*E*)-3-(4-{2-[4,4-dimethyl-1-(prop-2-yn-1-yl)-1,2,3,4-tetrahydro-quinolin-6-yl]ethynyl}phenyl)prop-2-enoate, DC473

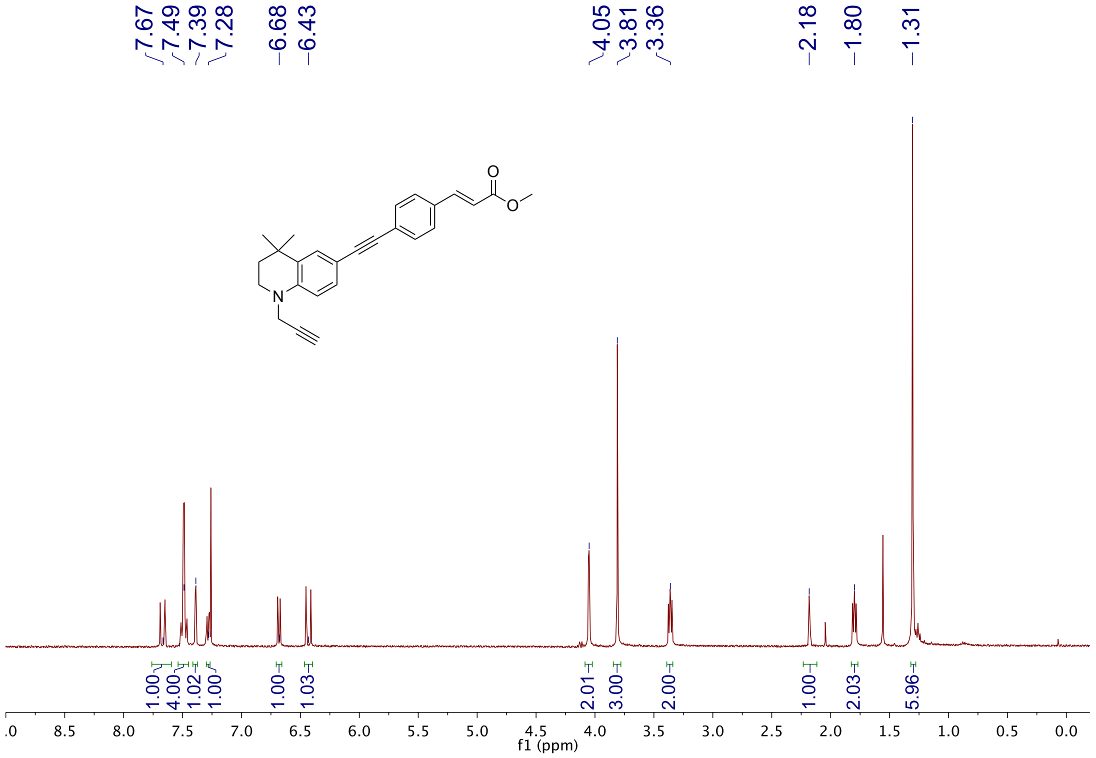

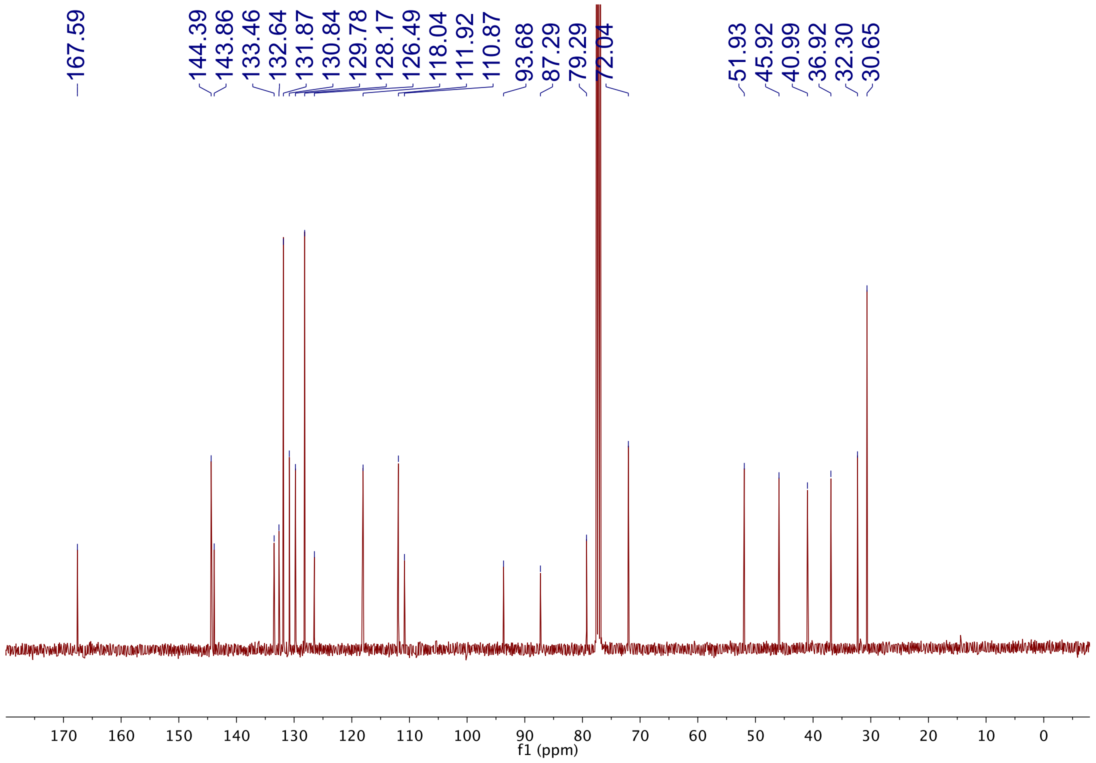

4. References

1 D. R. Chisholm, G.-L. Zhou, E. Pohl, R. Valentine and A. Whiting, *Beilstein J. Org. Chem.,* 2016, **12**, 1851-1862.

2 D. R. Chisholm, R. Lamb*,* T. Pallett, V. Affleck, C. Holden, J. Marrison, P. O'Toole, A. Steffen, A. Nelson, C. Mahler, J. Girkin, R. Valentine, T. B. Marder, A. Whiting and C. A. Ambler, *Chem. Sci.*, submitted.

3 E. C. Davison, I. T. Forbes, A. B. Holmes and J. A. Warner, *Tetrahedron*, 1996, **52**, 11601-11624.
